# Trends towards stronger primary care in three western European countries; 2006-2012

**DOI:** 10.1186/s12875-016-0458-3

**Published:** 2016-05-28

**Authors:** Tessa van Loenen, Michael J. van den Berg, Stephanie Heinemann, Richard Baker, Marjan J. Faber, Gert P. Westert

**Affiliations:** Scientific Institute for Quality of Healthcare, Radboud University Medical Center, 114 IQ healthcare, PO Box 9101, 6500 HB Nijmegen, The Netherlands; National Institute for Public Health and the Environment (RIVM), Bilthoven, The Netherlands; Department of Social Medicine, Academic Medical Center, Amsterdam, The Netherlands; Department of General Practice, University of Göttingen, Göttingen, Germany; Department of Health Sciences, University of Applied Sciences Fulda, Fulda, Germany; Department of Health Sciences, University of Leicester, Leicester, UK

**Keywords:** Primary health care, Healthcare systems, Continuity of care, Accessibility of care

## Abstract

**Background:**

Strong primary care systems are believed to have an important role in dealing with healthcare challenges. Strengthening primary care systems is therefore a common policy goal for many countries. This study aims to investigate whether the Netherlands, the UK and Germany have strengthened their primary care systems in 2006-2012.

**Method:**

For this cross-sectional study, data from the International Health Policy surveys of the Commonwealth Fund in 2006, 2009 and 2012 were used. The surveys represent the experiences and perspectives of primary care physicians with their primary care system. The changes over time were researched in three areas: organization of primary care processes, use of IT in primary care and use of benchmarking and financial incentives for performance improvement.

**Results:**

Regarding organization of primary care processes, in all countries the use of supporting personnel in general practice increased, but at the same time practice accessibility decreased. IT services were most advanced in the UK. The UK and the Netherlands showed increased use of performance feedback information. German GPs were least satisfied with how their system works across the 2006-2012 timeframe.

**Conclusion:**

All three countries show trends towards stronger primary care systems, although in different areas. Coordination and comprehensive care through the assignment of assisting personnel and use of disease management programs improved in all countries. In the Netherlands and the UK, informational continuity is in part ensured through better IT services. All countries showed increasing difficulties upholding primary care accessibility.

**Electronic supplementary material:**

The online version of this article (doi:10.1186/s12875-016-0458-3) contains supplementary material, which is available to authorized users.

## Background

Primary care is believed to have an important role in dealing with the healthcare challenges many countries face, including rising healthcare costs, the increasing prevalence of chronic conditions and multi-morbidity, health inequalities and potentially avoidable hospitalization [1, 2]. These challenges put enormous pressure on healthcare systems. Primary care is assumed to alleviate some of the pressure by increasing the population’s health at lower costs. Strengthening their primary care systems is therefore a common policy goal for many countries [3, 4]. Strong primary care systems provide care close to patients with minimal *access* barriers, meet the many needs of patients with *comprehensive* services, *coordinate* care through all healthcare levels and establish a doctor-patient relationship that is *continuous* over time [4, 5]. Strong primary care systems can therefore contribute to better patient care. This definition of strong primary care systems implies that the structure of primary care and the process of service delivery include a set of features and characteristics, which are summarized into four main domains: accessibility, comprehensiveness, continuity, and coordination. How these domains are operationalized in the organization of primary care systems depends substantially on country characteristics, historical background or health (care) problems [6]. For instance, this can be operationalized by introducing out-of-hours care in order to provide accessible care. On other example are IT services, which can help increase informational continuity and better coordination of care services.

In western European countries, most reforms aim to create sustainable healthcare systems focusing on efficiently organized primary care services. In many countries, for example, medical tasks are increasingly being delegated from GPs to nurses or other staff [7]. Also, accessibility of primary care services, including out-of-hours care, needs to be guaranteed in order to prevent unnecessary and more expensive secondary care or emergency department use [8]. Other improvements include modernizing processes by using information technology (IT) such as electronic medical records to support primary care organization. Better IT services can increase informational continuity of care when medical information about patients is available for all professionals treating the patient, especially when care is coordinated through different levels of the healthcare system. To establish such performance improvements, general practitioners (GPs) will need to be stimulated to change their practices and behaviour, for instance through extra financial incentives for specific services and benchmark information on quality outcomes.

The Netherlands, the UK and Germany are three western European countries that face common problems such as increasing health care spending and a changing demand for care, and recognize the potential beneficial role of strong primary care, although their policy measures differ. This can be understood given the historical differences in the design and positioning of primary care in the healthcare system. The UK has a public healthcare system (Beveridge) whereas the German healthcare system is mainly a social health insurance system (Bismarck). Since 2006, the Dutch health insurance system is semi-public, based on Enthovens’ model which incorporates managed competition. In the Netherlands and the UK, primary care is the centre of the healthcare system; GPs function as gatekeepers for secondary care and there is a patient list system, implying that all patients are assigned to a GP. In Germany there is a strong distinction between primary and secondary care, limited gatekeeping and absence of a patient list system [9].

This study aims to describe whether the Netherlands, the UK, and Germany strengthened the structure of their primary care systems by looking at three areas: organizational processes such as the use of support staff and out-of-hour’s arrangements, the use of IT services within primary care, and the use of incentives for performance improvement. We focused on those aspects in the organization of primary care that can help support primary care in providing accessible, coordinated, comprehensive and continuous care. The Commonwealth Fund International Health Policy data of 2006, 2009 and 2012 were used to present these changes over time [10–12]. In addition, we researched whether these changes were reflected in the experience of GPs with their healthcare system. The following paragraphs describe recent policy changes for the Netherlands, the UK and Germany relevant for this study.

### Primary care in the Netherlands

The Dutch primary care system is often referred to as the “spine” of Dutch healthcare. Almost 100 % of the Dutch citizens are registered with a general practice. GPs have a gatekeeping role; they are patient’s first point of contact, and referral is required for secondary care. There is a strict division between primary and specialty care. GP care in the Netherlands is available without co-payments. The majority of GPs used to work in small-scale practices, but the proportion of doctors working in partnership has been growing. At the moment, about half of the GPs are working in duo- or single-handed practices [13]. The number of assisting personnel in practices such as nurse practitioners and practice nurses is rising. In addition, the transition of tasks from the GP to these assisting personnel has been growing [14–16]. Dutch primary care is known for good accessibility during office hours but also after office hours, patients can receive primary care in out-of-hours GP cooperatives [14–16].

In 2006 a managed competition system based on Enthoven’s model was introduced for curative care. In this system, health insurers act as contracting parties. They can compete on prices, services, and the quality of care provided by the professionals they contracted [17, 18]. After the reform people were obligated to get individual health insurance from a private insurance company which provides an obligatory universal package including a basic set of medical treatment and services specified by the government [13]. As a consequence of this payment reform, Dutch primary care providers organized themselves in so-called care groups to provide integrated care for chronic patients. Health insurance companies can negotiate with the care group to settle a bundled payment contract.

### Primary care in the UK

In the UK (incorporating England, Wales, Scotland and Northern Ireland), most health care services are free at the point of delivery. Health care is funded through National Health Services (NHS). Responsibility for primary care services at the local level lay with NHS administrative organizations, primary care trusts, during the period of the study. All primary care is provided by general practitioners who are in most cases the point of access to care. GPs in the UK have a gatekeeping role. Patients can choose the practice with whom they register. In the past, most GP’s owned solo practices, nowadays there is an increasing trend towards larger group practices which on average have 4–6 physicians. Also the task substitution from physicians to nurses has been increasing [14, 19, 20].

As of 2004, a new GP contract was introduced with the intention to improve patient access to care and change payments for GPs based on their performance. The new contract included payments for essential services, enhanced services, out-of-hours care and the Quality and Outcome Framework (QOF). Within the QOF, practices are rewarded for achievements on a range of quality indicators. The reform forced practices to adapt the use of full electronic medical records, which enables the production of benchmarking information [14, 19, 20].

### Primary care system in Germany

Primary care in Germany has only recently begun to develop as the healthcare system traditionally concentrated on inpatient and specialist care. In 2012, 46 % of all practicing social health insurance (SHI) accredited physicians for ambulatory care were family physicians, whereas 54 % were specialists. Most ambulatory care is provided by general/family practitioners, general internists or paediatricians. Primary care physicians work mainly in single-handed or in small group practices. The care services are almost exclusively provided by a physician. Support staff who provide care, such as practice nurses or nurse practitioners, are uncommon in German primary care. Support staff in GP practices assist physicians mainly in administrative tasks [21, 22]. Since 2009, health insurance is mandatory, either through SHI or private health insurance. About 90 % are insured though SHI. German primary care physicians do not have a formal gate-keeping role. Sickness funds offer voluntary “family physician care models”, whereby patients register with a primary care physician and are required to go to this physician as first point of care. In exchange, sickness funds offer enrolled insured members special evening office hours, shorter waiting times for the GP and exemption from co-payments on some pharmaceuticals.

For patients, there is free choice and access to both primary and secondary health care services. Between 2004 and 2012, all SHI insured were required to pay user charges for SHI care. This practice fee (“Praxisgebühr”) cost 10€ once per quarter and was required for consulting SHI physicians, psychotherapists and dentists. This fee was not an attempt at gatekeeping, however, since patients were not required to go to a primary care physician first in order to gain access to secondary care. Rather, access at any point of the healthcare system (even to GPs) cost a flat fee of 10€ per quarter. This fee was then revoked in January 2013. Another important recent development includes the introduction of disease management programs starting 2003 in order to provide comprehensive care to chronically ill patients [21]. Pay-for-performance and benchmarking information are not instruments commonly used in German primary care, although auditing for prescription of pharmaceuticals and medical aids exceeding by more than 15 and/or 25 % the agreed reference volumes can result in regress (i.e. physicians have to pay a penalty for over-prescribing pharmaceuticals and/or medical aids).

## Methods

### Data sources

Data from the International Health Policy (IHP) study of the Commonwealth Fund were used. In 2006, 2009 and 2012 primary care physicians in several countries were asked to complete a questionnaire about their experiences of their healthcare system [10–12]. The respondents were chosen randomly from public or private lists of working primary care physicians in a country. The 2006 survey consisted of an interview by phone or a postal questionnaire sent to representative samples of primary care physicians in seven countries. In 2009 and 2012, the survey was conducted in 11 countries. The Netherlands, the UK and Germany participated in all three surveys. Whilst it would have been an advantage to have included other countries, no other European country took part in all three rounds.

In the Netherlands and the UK, only GPs participated. In Germany, paediatricians were also included as they provide primary care for children. All questionnaires were based on the previous survey, meaning they included several of the same questions and topics. Sample sizes varied between 500 and 1063 and the response rates ranged between 18 and 50 %. The representativeness of the sample was confirmed by comparison of the different samples to the initial characteristics available from the lists of physicians [10–12]. More details on the sample size, response rate and the characteristics of respondents in each country at each time point are presented in Table 1.

**Table 1:** Country and respondent characteristics

	Year	N	Response rate (%)	Gender *%female*	Age % *35–64*	Practice location *%City*	Practice size *%solo practices(1FTE)*	Density of GPs per 1000 population [34]^a^
UK	2006	1063	20	36.8	92.3	37.4	14	0.72
	2009	1062	20	38.3	83.0	26.9	12	0.79
	2012	500	24	39.0	85.0	37.8	8	0.80
Germany	2006	1006	18	38.2	90.1	49.8	68	0.66
	2009	715	50	38.2	91.9	24.4	50	0.65
	2012	909	20	37.7	91.5	42.0	53	0.67
Netherlands	2006	931	43	32.3	96.5	20.0	72	0.68
	2009	614	50	36.7	92.7	17.3	56	0.72
	2012	522	48	39.2	92.2	20.8	57	0.77

^a^note: these are head counts per 1000 population and not FTE. In Germany no paediatricians are included

Since the survey was non-medical, there was no ethical approval required from the Ethical Review Board. Written informed consents were not necessary. Participation in the survey was voluntary. Confidentiality was maintained by data coding to eliminate the identification of data with personal information.

### Variables

The analysis was based on three areas: organization of primary care, IT to support primary care processes and incentives for performance improvement. Organization of primary care was described by three variables: percentage of GPs where almost all patients can get an appointment the same or next day; the availability of out-of-hours arrangements; the mean full time equivalent (FTE) of non-physicians per 1 FTE physician.

The following variables for IT services to support processes were included: the percentage of GPs using electronic medical records; the possibility for GPs to send computerized reminder notices to patients who should receive care; the percentage that routinely get alerts for providing test results to patients; the ability to generate (a) a list of all patients by diagnosis or health risk; b) a list of patients due or overdue tests or preventive care; and, (c) a list of medications taken by individuals.

Variables that reflect incentives for performance improvement within the practice included extra financial incentives for chronic diseases or preventive care or routine feedback data on clinical outcomes or patient satisfaction.

Finally, a measure on the overall view of the healthcare systems was included. GPs were asked whether they thought the system worked well, needed changes or should be rebuilt.

More details on the variables can be found in Additional file 1.

### Statistical analyses

Descriptive statistics were used for analysing the different variables. The samples were weighted to represent age, sex, region and primary care speciality (only in Germany). The analyses were stratified per country. For the dichotomous outcome measure the differences in response between the years were tested with a chi-square test. One-way ANOVA was used to compare the means of the continuous outcomes measures over the 3 years. Data was analyzed with SPSS22. Statistical significance was assessed as *p* < 0.05. The results of the statistical comparison between countries can be found in Additional file 2.

## Results

The response rates per questions varied from 97.3 to 99.5 % in Germany, from 91.8 to 99.7 % in the Netherlands and from 95.4 to 99.8 % in the UK. Table 2 presents the proportions and means over time. There was a large significant increase in the number of non-physician personnel working per FTE physician in the practice. Particularly in Germany, physicians were increasingly supported by assisting personnel. Primary care accessibility after hours was already high in all countries in 2006. In 2012, about 90 % of the GPs stated that there were out-of-hours arrangements. However, all countries showed a significant decrease in the percentage of patients who could get an appointment on the same or the next day. While in the Netherlands and Germany, a decrease of around 10 % took place between 2006 and 2009, a decrease of 18 % in the UK between 2006 and 2012.

**Table 2:** Proportions and means

	Country	2006	2009	2012	Change 2006-2012 *- = decrease + = increase*
Organization of Primary care					
Non physician per FTE physician within practice	Germany	2.24	2.08*	2.87*‡	+ (*p* < 0.001)
	Netherlands	1.22	1.38*	1.70*‡	+ (*p* < 0.001)
	UK	0.92	1.04*	1.32*‡	+ (*p* < 0.001)
Out-of –hours care (%)	Germany	76.1	54.3*	89.6*‡	+ (*p* < 0.001)
	Netherlands	96.3	97.0	95.3	
	UK	87.1	88.8	95.5*‡	+ (*p* < 0.001)
Same or next day appointment (% almost all >80 %)	Germany	69.0	57.3*	56.6*	- (*p* < 0.001)
	Netherlands	71.9	62.1*	61.5*	- (*p* < 0.001)
	UK	73.4	64.8*	55.3*‡	- (*p* < 0.001)
IT to support organization of primary care					
Use electronic medical records (%)	Germany	42.1	73.3*	83.2*‡	+ (*p* < 0.001)
	Netherlands	97.9	99.8*	98.7‡	
	UK	89.5	96.8*	96.8*	+ (*p* < 0.001)
Reminder notices to patients receiving care (%)	Germany	27.6	17.4*	18.1*	- (*p* < 0.001)
	Netherlands	61.0	48.6*	43.1*	- (*p* < 0.001)
	UK	83.3	76.8*	65.5*‡	- (*p* < 0.001)
Alert/prompt for providing test results (%)	Germany	32.0	11.7*	11.3*	- (*p* < 0.001)
	Netherlands	16.5	7.8*	6.6*	- (*p* < 0.001)
	UK	53.5	49.2	58.3‡	
List of patients by diagnosis or health risk (% easy)	Germany	80.6	71.1*	55.6*‡	- (*p* < 0.001)
	Netherlands	63.3	67.1	78.1*‡	+ (*p* < 0.001)
	UK	92.5	97.3*	96.0*	+ (*p* = 0.009)
List of patients due or overdue for tests or preventive care (%easy)	Germany	63.7	39.0*	41.7*	- (*p* < 0.001)
	Netherlands	41.9	65.0*	73.0*‡	+ (*p* < 0.001)
	UK	77.2	90.9*	89.6*	+ (*p* < 0.001)
List of all medications taken by individual patients (%easy)	Germany	54.9	58.1	62.2*	+ (*p* = 0.001)
	Netherlands	59.8	70.0*	78.8*‡	+ (*p* < 0.001)
	UK	87.8	90.4	98.5*‡	+ (*p* < 0.001)
Incentives for performance improvement					
Incentive for patients with chronic diseases (%)	Germany	24.0	49.7*	61.0*‡	+ (*p* < 0.001)
	Netherlands	50.0	62.2*	77.6*‡	+ (*p* < 0.001)
	UK	80.5	84.8	51.7*‡	- (*p* < 0.001)
Incentive for enhanced preventive care (%)	Germany	28.3	24.9	23.3*	- (*p* = 0.01)
	Netherlands	19.0	17.8	28.8*‡	+ (*p* < 0.001)
	UK	74.2	39.4*	38.1*	- (*p* < 0.001)
Receives data on clinical outcomes (%)	Germany	70.9	41.3*	54.1*‡	- (*p* < 0.001)
	Netherlands	36.7	64.8*	82.0*‡	+ (*p* < 0.001)
	UK	78.1	90.8*	85.4*‡	+ (*p* = 0.001)
Receives data on patient satisfaction (%)	Germany	27.4	24.5	34.8*‡	+ (*p* = 0.001)
	Netherlands	19.1	22.7	39.7*‡	+ (*p* < 0.001)
	UK	89.5	96.7*	84.9*‡	- (*p* = 0.01)

*Significant (*p* < 0.05) change compared to 2006 ‡ Significant (*p* < 0.05) change between 2009 and 2012

All countries had at least 80 % of GPs using electronic medical records in 2012. With regards to the use of other IT services within the practice, there were large differences between countries. The UK scored high on all of the measures over the years, only showing a decrease of about 20 % on the possibility of sending reminder notices to patients when it is time for care. The Netherlands and Germany lagged behind in these developments. Germany showed a decrease of over 20 % on the possibility of making lists of patients or getting an alert for providing test results. The only increase shown in Germany was on the possibility of creating a list of medications that patients use.

In the Netherlands and Germany, the proportion of GPs who could receive incentives for patients with chronic diseases increased. In contrast, in the UK there was a strong decrease in incentives for patients with chronic diseases. The UK also showed a strong decrease in incentives for enhanced preventive care. The UK continued to score high on receiving clinical data and data on patient satisfaction. The Netherlands showed a strong increase on both variables, whereas Germany showed a strong decrease in receiving data about clinical outcomes.

**Fig. 1:**
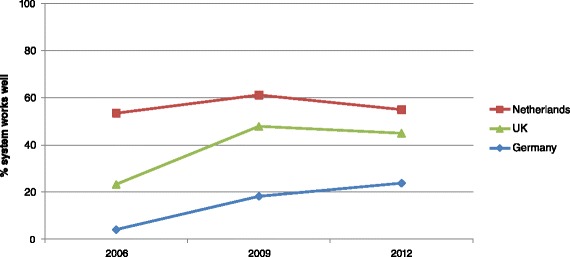
Percent GPs who thinks the healthcare system works well

Dutch GPs were most satisfied with how the system worked; over 50 % in all years indicated the system worked well (Fig. 1). GPs in Germany and the UK became more satisfied over the years. Both countries showed an increase of more than 20 %, although German GPs scored considerably lower compared to the other two countries.

## Discussion

The results of this study show that in all three countries major shifts took place within primary care. The use of support staff within general practice increased significantly. Despite this increase, the accessibility of primary care is under pressure. Accessibility is one of the main pillars of primary care, but GPs report that patients have to wait longer for an appointment during office hours. Accessibility of primary care for patients during out-of-hours showed to be very good in the three countries. It seems contradictory that accessibility during office hours is decreasing while it is increasing after office hours. One explanation is that out-of-hours care in the studied countries is arranged at the level of region and not by the individual practice. For instance, in the Netherlands primary care after office hours is arranged by GP cooperatives, where primary care out-of-hours services are run by GPs on a rotation basis. Regarding IT services in the practice, the use of electronic medical records has become fairly common. For other IT services that can support primary care, large differences between the countries exist. Practices in the UK are at the front of these developments. The UK increasingly uses feedback on performance and patient satisfaction, but getting additional incentives and money for certain services worsened. The Netherlands is focusing more on incentives for performance improvement, whereas in Germany these developments are lagging behind.

In all three countries, accessibility during office hours decreased. This finding probably reflects the increased pressure on GP services over the years. The demand for care has increased due to more chronic patients and multi morbidity, but the supply of GPs has remained static over the years. Two other surveys showed that mainly the general population experience difficulties accessing the primary care system rather than chronic patients [23, 24]. A study assessing the potential areas for primary care to improve based on patients’ perspectives showed that patients perceive the accessibility in the UK, the Netherlands and Germany as good [25]. Possibly, accessibility, although decreased, is still acceptable to most of the patients. Nevertheless, the decreasing accessibility during office hours deserves further investigation to see if this trend has continued and how this affect quality of care and patient outcomes.

The results of this survey show clear increases in Dutch practices receiving data on clinical outcomes as well as data on patient satisfaction. The importance of transparent and accessible quality information is recognized, especially since the introduction of the managed competition system in the Netherlands. Continuity of care is one of the cornerstones for future primary care in the Netherlands [26]. IT services are acknowledged to support informational continuity by establishing an efficient way of communication between GPs and patients. Although some of the IT opportunities have improved over the years, others such as the use of reminder notices and alerts for providing test results have decreased. So, the trend towards better informational continuity is only partly reflected in the results. Another trend in the Netherlands was the increased number of non-physicians working within the GP practice, often working part-time. This trend as well as the trend to increase the roles and competences of assisting personnel within general practices has been shown in previous studies. For instance, many practices have specialized nurses for treatment of patients with chronic conditions such as diabetes or COPD. Also since 2007, specialized nurses are allowed to prescribe medications in some instances [13]. In conclusion, the Netherlands has strengthened primary care by enhancing continuity through improvements in IT services, as well as the coordination and comprehensiveness of care for chronic patients.

In the UK, the introduction of the QOF in 2004 introduced rewards for practices that reach specific targets. Data on clinical outcomes can be used to measure the quality of care. Our results show that already in 2006, a large proportion of practices received feedback data on clinical outcomes as well as data on patient satisfaction. General practices in the UK often have an excellent multifunctional electronic health information system and seems to be in a more advanced state than in the other countries. Currently, the NHS is investing even more in extending high quality IT services in all practices [27]. Interesting is the decrease in possibility for GPs to receive extra incentives for chronic patient or preventive care. A study comparing GP services profiles from 1993 to 2014 showed that GPs in England are increasingly involved in disease management and the prevention activities stayed static over the years [28]. Nevertheless, we showed a drastic decrease in incentives. This study period includes the financial crisis. Data from the OECD show that during the financial crisis funding for health care stagnated or even decreased [29]. As a consequence, the funding in the primary care sector declined. Especially there have been cutbacks at local level in the local enhanced services (LES) [30, 31]. Overall, primary care in the UK scored already high on all elements of strong primary care in 2006, and the focus of the past years is mainly on improving and enhancing the quality of care.

Whereas the Netherlands and the UK have a long tradition of GP-centred primary care and gatekeeping, in Germany the development of mandatory first contact GP care is relatively new and only relevant for patients who are freely willing to enter a GP-centred care contract. One of the major changes in German primary care was the introduction of integrated care through disease management programs, in order to create comprehensive and well-coordinated care for the chronically ill. This resulted in a more team-oriented approach in which the role of nurses and practice assistants expanded [22, 32]. Indeed, the results show an increase in the use of support staff. In Germany, there has been a stagnated workforce of GPs, which makes the use of support staff in the practice even more important, considering the higher demand for care. Another consequence of the introduction of disease management programs is reflected in the number of GPs that can get extra incentives for treatment of patient with chronic conditions, which shows an upward trend. The use of electronic medical records in Germany doubled within the timeframe of 2006-2012, whereas the functional usage of IT in Germany lags behind. The inadequate development of the IT services in Germany compared to the UK and the Netherlands might be due to the large number of competing companies developing software for usage in local practices or the absence of a patient list system.

Interesting is how the shifts within primary care affects GPs’ satisfaction with their healthcare system. In the Netherlands the proportion of GPs that are satisfied with the system is consistently high. The question is whether we can expect it to increase or if this is the ceiling. As for the UK, there was a major increase in satisfaction between 2006 and 2009. Between 2009 and 2012, there was a small decline which might reflect the funding squeeze and increased demand for care. In Germany, there has been an impressive increase in satisfaction, but it is still lower than in the other two countries. The introduction of disease management programs and voluntary “family physician care models” have increased the role and the earnings of GPs in Germany, which may explain the increase in satisfaction with the healthcare system. However, a study from Behmann et al. in [33] looked into GP satisfaction rates and found that many GPs in Germany remain unsatisfied with both their earnings and the administrative burden of being a GP.

This study showed some changes in the structure of primary care in the Netherlands, the UK and Germany. Several of these changes may have affected the quality of care, patient satisfaction and patient outcomes, both in a positive and in a negative way. Future research should shed a light on the effects of such structural changes.

### Strengths and limitations

The strength of this study lies in the 3-year cycle of the survey, covering a timeframe of 6 years. Such a long follow-up enables identification of possible effects of reforms as perceived by GPs. Limitations of this study lie in the different methods of data collection between the countries and years, which might have introduced response bias. In the UK, all data were obtained primarily by telephone interviews, whereas in the Netherlands data were collected using postal paper-based surveys. In Germany, data were collected mainly by telephone interviews in 2006, but in the other years, postal surveys were used. Another limitation concerns wide variation in response rates between countries and between years. However, representative sampling was always confirmed: comparison of the different samples to the initial characteristics available from the lists of physicians showed no divergences [10–12]. Still, because of the differences in data collection and response rates, unknown bias can be introduced and interpreting the data should be done with caution.

## Conclusions

In the three countries, policy reforms have different focuses and consequences. Important is that all countries have a different starting point. The UK and the Netherlands, although having a different healthcare system, traditionally focus on GP-led primary care as the centre of the healthcare system. In Germany, primary care is more fragmented (e.g. paediatricians and GPs both provide primary care for children) and characterized by free choice of physician for patients. From 2006 to 2012, in all three countries a trend towards better *coordination* and *comprehensiveness* in primary care by use of assisting personnel and disease management programs is presented. Informational c*ontinuity* is in part ensured in the Netherlands and the UK through better IT services. The German primary care system can get stronger by establishing a better IT system within the practice but also by creating a system to assess quality and patient satisfaction. These improvements might also lead to better GP satisfaction. GPs in all three countries experienced difficulties keeping primary care directly *accessible* during office hours; out-of-hours care is well established. Even though all countries show developments towards stronger primary care systems, there are areas that need attention, such as the reduced accessibility to care. Keeping primary care accessible is crucial in an era of austerity, to avoid that patients show up at much more expensive spots in the healthcare system.

## Abbreviations

COPD, chronic obstructive pulmonary disease; FTE, full time equivalent; GP, general practitioner; IT, Information Technology; LES, local enhanced services; OECD, Organization for Economic Co-operation and Development; QOF, quality and outcomes framework; UK, United Kingdom.

## Additional files

Additional file 1: Detailed information on the variables. (DOCX 15 kb) Additional file 2: Proportions and means. Significance calculated between countries. (DOCX 18 kb)
