# Supplementary material for: Trends towards stronger primary care in three western European countries; 2006-2012
Source: BMC Fam Pract. 2016 May 28;17:59. doi: 10.1186/s12875-016-0458-3 (PMC4884410; doi:10.1186/s12875-016-0458-3)
Supplement: Additional file 1: — Detailed information on the variables. (DOCX 15 kb) [file 12875_2016_458_MOESM1_ESM.docx]

**Additional file 1. Detailed information on the variables**

|  | **Measures** |
| --- | --- |
| **Organization of Primary Care** |  |
| Ratio non physicians- physicians  *How many non-physician FTE health care providers (nurses, therapists or other clinicians) are in your practice?*  *How many full time equivalent (FTE) doctors, including yourself, are in your practice?* | Continuous |
| Out-of-hours care  *Does your practice have an arrangement where patients can see a doctor or nurse if needed when the practice is closed (after-hours) without going to the hospital emergency room or department?* | % Yes |
| Same or next day appointments  *What proportion of your patients who request a same- or next-day appointment can get one?* | % almost all (>80%) |
| **IT to support the PC organization** |  |
| Use of electronic medical records  *Do you use electronic patient medical records in your practice (not including billing systems)?* | %Yes |
| Computerized reminder notices to patients for receiving care  *Are the following tasks routinely performed in your practice? Patients are sent reminder notices when it is time for regular preventive or follow-up care (e.g., flu vaccine or HbA1C for diabetic patients)* | % Yes |
| Computerized alert/prompt for providing test results  *Are the following tasks routinely performed in your practice? You receive an alert or prompt to provide patients with test results* | % Yes |
| List of patients by diagnosis or health risk  *With the patient medical records system you currently have, how easy would it be for you (or staff in your practice) to generate the following information about your patients? Is this process computerized? List of patients by diagnosis (e.g., diabetes or cancer)* | % easy to generate |
| List of patients due or overdue for tests or preventive care  *With the patient medical records system you currently have, how easy would it be for you (or staff in your practice) to generate the following information about your patients? Is this process computerized? List of patients who are due or overdue for tests or preventive care (e.g., flu vaccine due)* | % easy to generate |
| List of all medications taken by individual patients  *With the patient medical records system you currently have, how easy would it be for you (or staff in your practice) to generate the following information about your patients? Is this process computerized? List of all medications taken by an individual patient (including those that may be prescribed by other doctors)* | % easy to generate |
| **Incentives for performance improvement** |  |
| Receives data on clinical outcomes  *Does the place where you practice routinely receive and review data on the following aspects of your patients’ care? Clinical outcomes (e.g., percent of diabetics or asthmatics with good control)* | % Yes |
| Receives data on patient satisfaction  *Does the place where you practice routinely receive and review data on the following aspects of your patients’ care? Surveys of patient satisfaction and experiences with care* | % Yes |
| Incentive for patients with chronic diseases  *Do you have the potential to receive or do you receive extra financial support based on any of the following? (This includes special payments, higher fees, or reimbursements.) Managing patients with chronic disease or complex needs* | % Yes |
| Incentive for enhanced preventive care  *Do you have the potential to receive or do you receive extra financial support based on any of the following? (This includes special payments, higher fees, or reimbursements.) Providing enhanced preventive care activities, including patient counseling or group visits* | % Yes |
| **Overall** |  |
| Overall view of the healthcare system  *Which of the following statements comes closest to expressing your overall view of the healthcare system in your country? Please select one.*  *- On the whole the healthcare system works pretty well and only minor changes are necessary to make it work better.*  *- There are some good things in our health system, but fundamental changes are needed to make it work better.*  *- Our healthcare system has so much wrong with it that we need to completely rebuild it.* | % system works well |
