# Supplementary material for: Trends towards stronger primary care in three western European countries; 2006-2012
Source: BMC Fam Pract. 2016 May 28;17:59. doi: 10.1186/s12875-016-0458-3 (PMC4884410; doi:10.1186/s12875-016-0458-3)
Supplement: Additional file 2: — Proportions and means. Significance calculated between countries. (DOCX 18 kb) [file 12875_2016_458_MOESM2_ESM.docx]

**Additional file 2. Proportions and means. Significance calculated between countries**

|  | **Country** | **2006** | **2009** | **2012** |
| --- | --- | --- | --- | --- |
| **Organization of Primary care** |  |  |  |  |
| Non physician per FTE physician within practice | Germany | 2.24 | 2.08 | 2.87 |
|  | Netherlands | 1.22  ‡(p<0.001) | 1.38  ‡(p<0.001) | 1.70  ‡(p<0.001) |
|  | UK | 0.92  ‡(p<0.001)  *(p<0.001) | 1.04  ‡(p<0.001)  *(p<0.001) | 1.32  ‡(p<0.001)  *(p<0.001) |
| Out-of –hours care  (%) | Germany | 76.1 | 54.3 | 89.6 |
|  | Netherlands | 96.3  ‡(p<0.001) | 97.0  ‡(p<0.001) | 95.3  ‡(p<0.001) |
|  | UK | 87.1  ‡(p<0.001)  *(p<0.001) | 88.8  ‡(p<0.001)  *(p<0.001) | 95.5  ‡(p<0.001) |
| Same or next day appointment  (% almost all >80%) | Germany | 69.0 | 57.3 | 56.6 |
|  | Netherlands | 71.9 | 62.1 | 61.5 |
|  | UK | 73.4  ‡(p=0.028) | 64.8  ‡(p=0.001) | 55.3  *(p=0.046) |
| **IT to support organization of primary care** |  |  |  |  |
| Use electronic medical records (%) | Germany | 42.1 | 73.3 | 83.2 |
|  | Netherlands | 97.9  ‡(p<0.001) | 99.8  ‡(p<0.001) | 98.7  ‡(p<0.001) |
|  | UK | 89.5  ‡(p<0.001)  *(p<0.001) | 96.8  ‡(p<0.001)  *(p<0.001) | 96.8  ‡(p<0.048)  *(p<0.001) |
| Reminder notices to patients receiving care (%) | Germany | 27.6 | 17.4 | 18.1 |
|  | Netherlands | 61.0  ‡(p<0.001) | 48.6  ‡(p<0.001) | 43.1  ‡(p<0.001) |
|  | UK | 83.3  ‡(p<0.001)  *(p<0.001) | 76.8  ‡(p<0.001)  *(p<0.001) | 65.5  ‡(p<0.001)  *(p<0.001) |
| Alert/prompt for providing test results (%) | Germany | 32.0 | 11.7 | 11.3 |
|  | Netherlands | 16.5  ‡(p<0.001) | 7.8  ‡(p=0.015) | 6.6  ‡(p=0.003) |
|  | UK | 53.5  ‡(p<0.001)  *(p<0.001) | 49.2  ‡(p<0.001)  *(p<0.001) | 58.3  ‡(p<0.001)  *(p<0.001) |
| List of patients by diagnosis or health risk (% easy) | Germany | 80.6 | 71.1 | 55.6 |
|  | Netherlands | 63.3  ‡(p<0.001) | 67.1 | 78.1  ‡(p<0.001) |
|  | UK | 92.5  ‡(p<0.001)  *(p<0.001) | 97.3  ‡(p<0.001)  *(p<0.001) | 96.0  ‡(p<0.001)  *(p<0.001) |
| List of patients due or overdue for tests or preventive care (%easy) | Germany | 63.7 | 39.0 | 41.7 |
|  | Netherlands | 41.9  ‡(p<0.001) | 65.0  ‡(p<0.001) | 73.0  ‡(p<0.001) |
|  | UK | 77.2  ‡(p<0.001)  *(p<0.001) | 90.9  ‡(p<0.001)  *(p<0.001) | 89.6  ‡(p<0.001)  *(p<0.001) |
| List of all medications taken by individual patients (%easy) | Germany | 54.9 | 58.1 | 62.2 |
|  | Netherlands | 59.8  ‡(p=0.029) | 70.0  ‡(p<0.001) | 78.8  ‡(p<0.001) |
|  | UK | 87.8  ‡(p<0.001)  *(p<0.001) | 90.4  ‡(p<0.001)  *(p<0.001) | 98.5  ‡(p<0.001)  *(p<0.001) |
| **Incentives for performance improvement** |  |  |  |  |
| Incentive for patients with chronic diseases (%) | Germany | 24.0 | 49.7 | 61.0 |
|  | Netherlands | 50.0  ‡(p<0.001) | 62.2  ‡(p<0.001) | 77.6  ‡(p<0.001) |
|  | UK | 80.5  ‡(p<0.001)  *(p<0.001) | 84.8  ‡(p<0.001)  *(p<0.001) | 51.7  ‡(p=0.001)  *(p<0.001) |
| Incentive for enhanced preventive care (%) | Germany | 28.3 | 24.9 | 23.3 |
|  | Netherlands | 19.0  ‡(p<0.001) | 17.8  ‡(p=0.002) | 28.8  ‡(p=0.022) |
|  | UK | 74.2  ‡(p<0.001)  *(p<0.001) | 39.4  ‡(p<0.001)  *(p<0.001) | 38.1  ‡(p<0.001)  *(p=0.002) |
| Receives data on clinical outcomes (%) | Germany | 70.9 | 41.3 | 54.1 |
|  | Netherlands | 36.7  ‡(p<0.001) | 64.8  ‡(p<0.001) | 82.0  ‡(p<0.001) |
|  | UK | 78.1 ‡(p<0.001)  *(p<0.001) | 90.8  ‡(p<0.001)  *(p<0.001) | 85.4  ‡(p<0.001) |
| Receives data on patient satisfaction (%) | Germany | 27.4 | 24.5 | 34.8 |
|  | Netherlands | 19.1  ‡(p<0.001) | 22.7 | 39.7 |
|  | UK | 89.5  ‡(p<0.001)  *(p<0.001) | 96.7  ‡(p<0.001)  *(p<0.001) | 84.9  ‡(p<0.001)  *(p<0.001) |

**‡ Significant difference (p<0.05) compared to Germany * Significant difference (p<0.05) compared to Netherlands**
